# Emodin Alleviates High-Glucose-Induced Pancreatic *β*-Cell Pyroptosis by Inhibiting NLRP3/GSDMD Signaling

**DOI:** 10.1155/2022/5276832

**Published:** 2022-02-27

**Authors:** Yiqian Xing, Yuchi He, Yuan Zhang, Heting Wang, Sihan Peng, Chunguang Xie, Jian Kang, Ya Liu, Xiyu Zhang

**Affiliations:** ^1^Chengdu University of Traditional Chinese Medicine, Chengdu 610072, China; ^2^First Affiliated Hospital of Army Medical University, Chongqing 400038, China; ^3^Department of Traditional Chinese Medicine, Sichuan Provincial People's Hospital, University of Electronic Science and Technology of China, Chengdu 610072, China; ^4^TCM Regulating Metabolic Diseases Key Laboratory of Sichuan Province, Hospital of Chengdu University of Traditional Chinese Medicine, Chengdu 610072, China; ^5^Department of Anorectal, Hospital of Chengdu University of Traditional Chinese Medicine, Chengdu 610072, China; ^6^Department of Endocrinology, Hospital of Chengdu University of Traditional Chinese Medicine, Chengdu 610072, China

## Abstract

Diabetes mellitus (DM) is a chronic noninfectious disease that is mainly featured by pancreatic *β*-cell (*β*-cell) dysfunction and impaired glucose homeostasis. Currently, the pathogenesis of dysfunction of the *β*-cells in DM remains unclear, and therapeutic approaches to it are limited. Emodin (EMD), a natural anthraquinone derivative, has been preliminarily proven to show antidiabetic effects. However, the underlying mechanism of EMD on *β*-cells still needs to be elucidated. In this study, we investigated the protective effects of EMD on the high glucose (50 mM)-induced INS-1 cell line and the underlying mechanism. INS-1 cells were treated with EMD (5, 10, and 20 *μ*M) when exposed to high glucose. The effects of EMD were examined by using the inverted phase-contrast microscope, qRT-PCR, ELISA, and western blot. The results showed that EMD could alleviate cellular morphological changes, suppress IL-1*β* and LDH release, and promote insulin secretion in high-glucose-induced INS-1 cells. Furthermore, EMD inhibits NOD-like receptor protein 3 (NLRP3) activation and gasdermin D (GSDMD) cleavage to alleviate pyroptosis induced by high glucose. Overexpression of NLRP3 reversed the above changes caused by EMD. Collectively, our findings suggest that EMD attenuates high-glucose-induced *β*-cell pyroptosis by inhibiting NLRP3/GSDMD signaling.

## 1. Introduction

Diabetes mellitus (DM), a severe disease with epidemic spreading throughout the world, is characterized by hyperglycemia. As of 2019, the number of people with DM has reached a total of 463 million. It is estimated that the number will reach 578 million by the year of 2030 and 700 million by 2045 [1]. Driven by the lack of insulin or ineffective production of insulin in the pancreas, high blood sugar gives rise to many life-threatening diabetic complications and makes DM a leading cause of cardiovascular morbidity and mortality, renal failure, amputations, and blindness [2]. *β*-Cells are pivotal for the maintenance of blood glucose homeostasis. In particular, they are the only source of insulin in humans; thus, they are a prime target in DM. Many synthetic drugs have been developed to decrease hyperglycemia and preserve *β*-cell function, but still, a complete cure is not provided by any of the molecules. Besides, continuous use of some synthetic agents may cause adverse side effects. Therefore, it is imperative to search for new compounds with therapeutic effects on *β*-cells.

**Figure 1:**
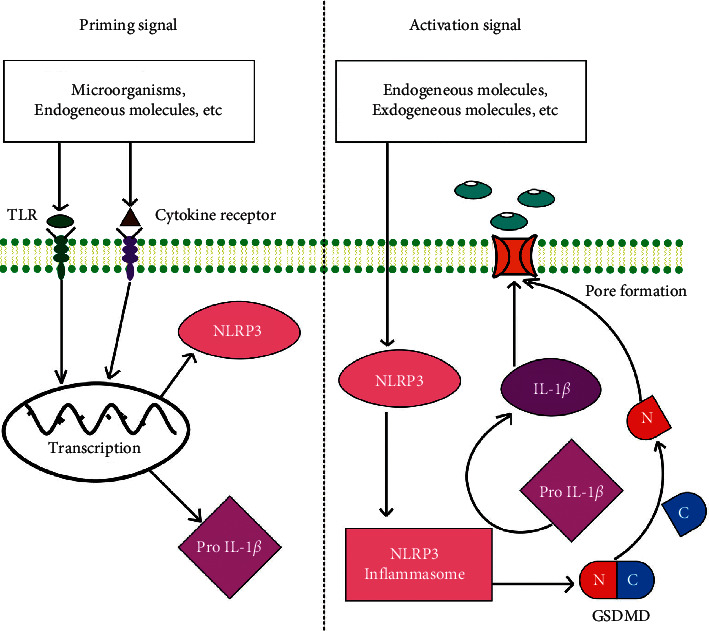
Model for two parallel pathways to activate NLRP3.

Pyroptosis is a unique type of inflammatory cell death featured in the cleavage of gasdermin D (GSDMD), which is caused by inflammasome activation [3]. The best defined inflammasome is the NOD-like receptor family pyrin domain-containing 3 (NLRP3) inflammasome. NLRP3 serves as an intracellular receptor of the NLRP3 inflammasome [4]. Activation of NLRP3 requires two parallel signaling pathways, priming and activation (Figure 1). Priming signal initiates the transcription of NLRP3 protein and the intracellular proform of IL-1*β* in response to toll-like receptors (TLRs) or cytokine receptors [5, 6]. Activation signal generates DNA fragmentation, cell membrane pore formation, cellular swelling with big bubbles, and release of mature interleukin-1*β* and other cytokines [5]. Increasing evidence shows that the pyroptosis activated by NLRP3 plays a vital role in the development of diabetes [7]. NLRP3 activation has been reported in *β*-cells, accompanied with IL-1*β* production and immune cell infiltration [8]. However, whether NLRP3 activation is involved in the *β*-cell destruction during chronic hyperglycemia has not reached a conclusion.

EMD (1,3,8 trihydroxy-6-methylanthraquinone) is an anthraquinone derivative extracted mainly from the root and rhizome of plant *Rheum palmatum*. This monomer has shown multiple pharmacological functions, such as anti-inflammation, immune suppression, antifibrotic, antioxidant, antitumor, and antidiabetic, among which the antidiabetic activity is prominent [9, 10]. EMD controls glucose homeostasis as a potent and selective 11*β*-HSD1 inhibitor [11]. The high GLP-1 secretion [12], inhibition of DPP-4 [13], IL-1*β* release [14], and glycogen synthase kinase-3*β* [15] expression caused by EMD demonstrated its potential to protect *β*-cells. Several attempts have been made to find its molecular mechanism. Recently, Liu et al. indicated EMD inhibited NLRP3 inflammasome activation and downregulated the level of IL-1*β* in the LPS-induced acute lung injury rat model and in J774A.1 cells [16]. In addition, Ye et al. found that EMD inhibited GSDMD expression and prevented ischemia-/reperfusion-induced pyroptosis in cardiomyocytes [17]. Meanwhile, emodin could protect acetaminophen-induced hepatotoxicity via the inhibition of NLRP3 activation in C57BL/6 mice [18] and protect pancreatic ductal cells against ATP-induced acute pancreatitis through the inhibition of the P2 × 7/NLRP3 signaling pathway [19]. Moreover, emodin was indicated to alleviate severe acute pancreatitis-associated acute lung injury through regulating the NLRP3/IL-1*β*/CXCL1 signaling [20]. Combining these findings, EMD demonstrated a potent antipyroptotic effect in several diseases including cardiomyopathy, pancreatitis, and pancreatitis-associated lung injury [21–24]. However, the effects of EMD on pyroptosis of *β*-cells remain unclear.

Therefore, in this study, we evaluated the effects of EMD on INS-1 cells induced by high glucose and whether EMD could inhibit *β*-cell pyroptosis via the NLRP3/GSDMD pathway.

## 2. Materials and Methods

### 2.1. Cell Culture and Drug Treatment

INS-1 cells, a rat pancreatic islet *β*-cell line, were obtained from the Institute of Basic Medical Sciences, Chinese Academy of Medical Sciences, Beijing, China. INS-1 cells were cultured in the RPMI-1640 medium (Procell Life Science and Technology Co., Ltd., Wuhan, China) containing 10% fetal bovine serum (Gibco, Australia), 10 mM HEPES, 5.5 mM glucose, 50 *μ*M *β*-mercaptoethanol, and 1% penicillin/streptomycin at 37°C in a humidified and aseptic incubator under 5% CO_2_ conditions. The culture medium was changed every three days.

EMD was purchased from Must Bio-Technology Company Limited (Chengdu, China). The purity of EMD was more than 98% (lot no. must-20092210). The concentration of EMD was 5, 10, and 20 *μ*M. INS-1 cells were seeded in 6-well plates when 90% confluence. Then, cells were randomly divided into 5 groups, respectively, with the following interventions: (1) normal control (NC) group (cells were cultured in normal conditions without any interventions); (2) high glucose (HG) group (to mimic the diabetic environment in vitro, cells were cultured in the aforementioned RPMI-1640 conditioned medium with 50 mM glucose for 48 h); (3, 4, 5) HG + EMD (5, 10, and 20 *μ*M) groups (cells were treated with HG and EMD treatment for 48 h). Lastly, the INS-1 cells were harvested for subsequent experiments.

### 2.2. Cell Morphology under the Inverted Phase-Contrast Microscope

An inverted phase-contrast microscope (DMi1, Leica, Germany) was used to observe the cellular morphological changes of INS-1 cells after each treatment.

### 2.3. LDH Release and Enzyme-Linked Immunosorbent Assay

Lactate dehydrogenase (LDH) is a soluble cytoplasmic enzyme that is present in almost all living cells and is released into the extracellular space when the cell membranes are damaged. Therefore, release of LDH was used as a biomarker for cellular cytotoxicity and cytolysis. LDH activity was measured using an LDH cytotoxicity assay kit (Solarbio Science and Technology Co., Ltd., Beijing, China) in accordance with the manufacturer's protocol.

The interleukin-1*β* (IL-1*β*) and insulin levels in the cell culture were detected using Rat IL-1*β* ELISA Kit (MultiSciences (Lianke) Biotechnology Co., Ltd., Hangzhou, China) and Rat Insulin Competitive ELISA Kit (MultiSciences (Lianke) Biotechnology Co., Ltd., Hangzhou, China) according to their instructions. Absorbance at a wavelength of 450 nm was detected. Standard curves were applied to calculate the concentrations of IL-1*β* and insulin. This experiment was conducted in triplicate.

### 2.4. Quantitative Real-Time PCR

Total RNA from INS-1 cells was extracted using RNA TRIzol reagent (Bomei Biotechnology Co., Ltd., Hefei, China) and reverse-transcribed to cDNA with TB Green Premix Ex TaqII (Takara, Dalian, China). The qRT-PCR was performed with SYBR Green using Thermo Scientific PikoReal (Thermo, USA) by predenaturing at 95°C for 30 s, denaturing at 95°C for 5 s, annealing at 55°C for 30 s, and extending at 72 C for 30 s. The primer sequences were as follows: NLRP3 forward (5′AAGCTGGCCCAGTATCTAGAGGAC-3′) and reverse (5′CCAAGTGATCTGCCTTCTCCATCTG-3′), GSDMD forward (5′TTCAAGCCCTACTGCCTCCTGAG-3′) and reverse (5′AGACACTGGTTCTGGAGCACTGG-3′), and ACTB forward (5′-CATCACTATCGGCAATGAGCGGTTCC-3′) and reverse (5′-ACGCAGCTCAGTAACAGTCCGCCTA-3′). The relative gene expression was calculated by the 2^−△△CT^ method using ACTB as the internal control.

### 2.5. Western Blot

Total protein of INS-1 cells was extracted and lysed in RIPA lysis buffer (Beyotime, Shanghai, China) including the protease inhibitor (Roche, CA, USA). A BCA protein assay kit (Beyotime, Shanghai, China) was used to quantify proteins, and samples were mixed with 1 SDS sample buffer (50 mM Tris, pH 6.8, 2% SDS, 10% glycerol, 50 mM DTT, and 0.01% bromophenol blue). Proteins were separated in 12% SDS-PAGE, transferred onto the PVDF membrane, and immunoblotted with anti-GSDMD (1 : 1000, ABclonal, USA) and anti-NLRP3 (1 : 1000, ABclonal, USA) at 4°C overnight. Secondary antibodies (goat anti-rabbit IgG H&L (HRP), Abcam, USA) were applied for 2-3 h at room temperature, and membranes were developed via the BCA protein colorimetric assay kit (Beyotime, Shanghai, China). Developed protein bands were quantified by Tanon Gel Image System V2.0 program (Shanghai, China).

### 2.6. Construction of the NLRP3 Overexpression Plasmid and Cell Transfection

The full-length NLRP3 sequence was constructed into empty plasmid (pcDNA), and these reconstructed plasmids were named pcDNA-NLRP3 (NLRP3). pcDNA was employed as the negative control. Cells were inoculated in a 6-well plate at a density of 2 × 10^5^/well. When cell confluence reached 90%, cells were transfected with different plasmids using the Lipofectamine 2000 kit (Invitrogen Inc., Carlsbad, California, USA). The target plasmids (2.5 *μ*g) and Lipofectamine 2000 (8 *μ*L) were separately diluted with 100 *μ*L serum-free Opti-MEM (Gibco Company, NY, USA). The two dilutions were allowed to stand at room temperature for 5 min and mixed evenly. The mixture was added to the culture well after being placed for 5 min and then cultured in an incubator with 5% CO_2_ at 37°C. The cells were randomly divided into 6 groups: (1) NC group; (2) HG group; (3) HG + pcDNA group (cells were transfected with pcDNC and then treated with HG for 48 h); (4) HG + NLRP3 group (cells were transfected with NLRP3 and then treated with HG for 48 h); (5) HG + pcDNA + EMD group (cells were transfected with pcDNA and then treated with HG and EMD (20 *μ*M) for 48 h); (6) HG + NLRP3 + EMD group (cells were transfected with NLRP3 and then treated with HG and EMD (20 *μ*M)for 48 h). Lastly, the INS-1 cells were harvested for subsequent experiments.

### 2.7. Statistical Analysis

Data were reported as the mean ± SD. The differences between two groups were compared by unpaired Student's *t*-tests, and one-way analysis of variance (ANOVA) was used for differences among groups with GraphPad Prism 8. Statistical differences were set at *P* < 0.05.

## 3. Results

### 3.1. High Glucose Induced Pyroptosis in INS-1 Cells

**Figure 2:**
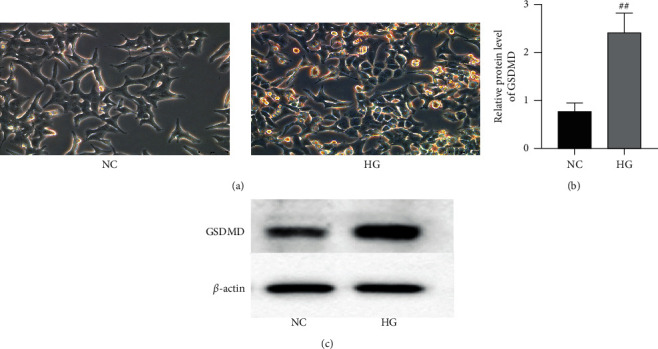
High glucose induced pyroptosis in INS-1 cells. (a) INS-1 cells were incubated with normal glucose for 48 h, and morphology was observed under the inverted phase-contrast microscope (×200). INS-1 cells were incubated with high glucose for 48 h, and the cellular swelling, irregular shrinkage patterns, and formations of closed-cell foams or bubbles appeared. (b) The protein level of pyroptosis-related protein GSDMD was measured by western blot in INS-1 cells. (c) *β*-Actin was used as the internal control. All data were shown as mean ± SD (*n* = 3), which were three independent experiments performed in triplicate. ^#^*P* < 0.05 and ^##^*P* < 0.01 were considered statistically significant.

INS-1 cells were observed under the inverted phase-contrast microscope (Figure 2(a)). In the NC group, INS-1 cells showed irregular shapes and sizes. In the HG group, the morphology of INS-1 cells changed after high glucose stimulation. Irregular shrinkage patterns and formations of closed-cell foams or bubbles appeared, and necrosis significantly increased. In addition, the pyroptosis-executed protein GSDMD expression was detected by western blot (Figures 2(b) and 2(c)). Compared with the NC group, the GSDMD protein was significantly elevated by high glucose stimulation *P* < 0.01, suggesting that high glucose induced INS-1 pyroptosis.

### 3.2. EMD Inhibited High-Glucose-Induced Injury in INS-1 Cells

**Figure 3:**
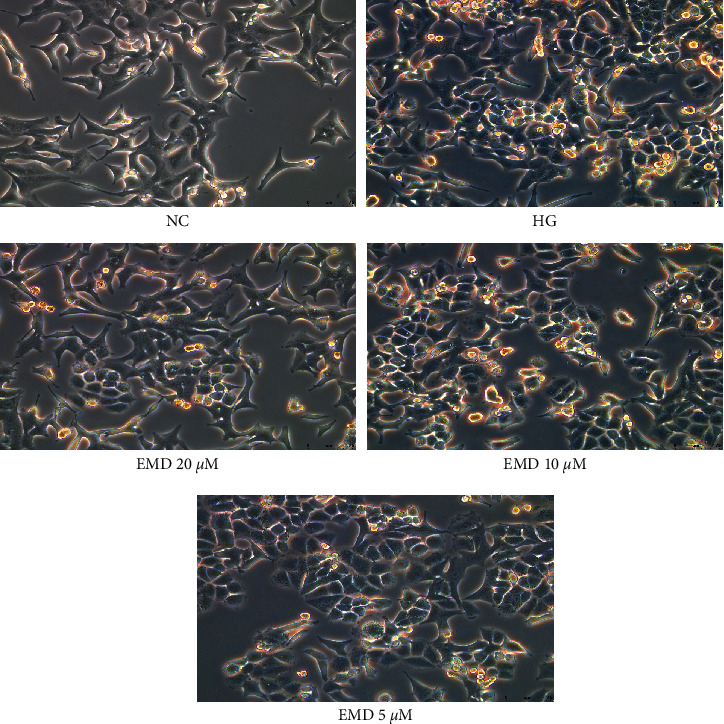
EMD attenuates the morphological changes induced by high glucose in INS-1 cells. The morphology is observed under the inverted phase-contrast microscope (×200).

We evaluated the effect of EMD on high-glucose-induced INS-1 cell pyroptosis. High glucose dramatically changed the morphology of INS-1 cells. By contrast, EMD treatment (5, 10, and 20 *μ*M) attenuated the morphological changes and preserved the INS-1 cells in normal shape during high glucose, which was in a dose-dependent manner (Figure 3).

**Figure 4:**
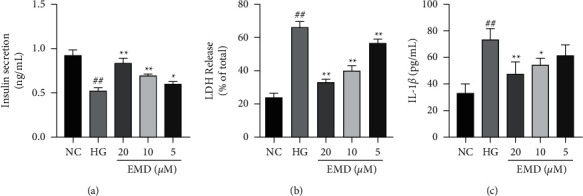
EMD promotes insulin secretion and decreases LDH and IL-1*β* levels in high-glucose-induced INS-1 cells. (a) Insulin levels are measured by the ELISA assay. (b) LDH levels are measured by the ELISA assay. (c) IL-1*β* levels are measured by the ELISA assay. Data are expressed as mean ± SD of three independent experiments. ^#^*P* < 0.05 and ^##^*P* < 0.01 vs. the NC group; ^*∗*^*P* < 0.05 and ^*∗∗*^*P* < 0.01 vs. the HG group.

Then, insulin secretion and the release of LDH were detected by ELISA in INS-1 cells (Figures 4(a) and 4(b)). Compared with the NC group, high glucose significantly decreased the insulin secretion and elevated the LDH level (all *P* < 0.01). However, EMD treatment (5, 10, and 20 *μ*M) rescued the loss of insulin-secreting ability in high-glucose-induced INS-1 cells (*P* < 0.05) and LDH leakage (*P* < 0.01) in a dose-dependent manner.

IL-1*β*, a proinflammatory cytokine, plays a crucial role in inflammation and pyroptosis, which is involved in dysfunction and destruction of *β*-cells. Thus, ELISA assay was performed to investigate the level of IL-1*β* (Figure 4(c)). Compared with the control group, high glucose significantly elevated IL-1*β* (*P* < 0.01), whereas EMD treatment (10 and 20 *μ*M) dramatically suppressed the IL-1*β* release (*P* < 0.05). These results indicated that EMD may inhibit inflammation and pyroptosis in high-glucose-induced INS-1 cells, which was in a dose-dependent manner. In addition, EMD at high concentration manifested the best effect to suppress pyroptosis in high-glucose-induced INS-1 cells. Therefore, EMD (20 *μ*M) was selected in subsequent experiments.

### 3.3. EMD Downregulated the Expression of Pyroptosis-Related Protein

**Figure 5:**
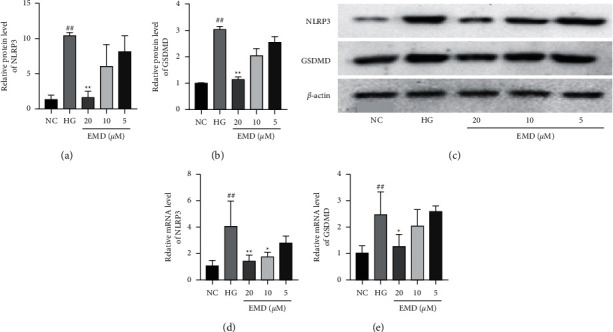
EMD downregulates NLRP3 and GSDMD expressions in high-glucose-induced INS-1 cells. (a, b) NLRP3 protein levels are measured by western blot. (c, d) GSDMD protein levels are measured by western blot. (e, f) NLRP3 and GSDMD mRNA levels are measured by qRT-PCR. Data are expressed as mean ± SD of three independent experiments. ^#^*P* < 0.05 and ^##^*P* < 0.01 vs. the NC group; ^*∗*^*P* < 0.05 and ^*∗∗*^*P* < 0.01 vs. the HG group.

Pyroptosis is initiated by NLRP3 activation and executed by a pyroptosis-related protein GSDMD. Therefore, western blot and qRT-PCR were performed to corroborate whether EMD treatment could downregulate the expression of NLRP3 and GSDMD in INS-1 cells (Figure 5). Compared to the NC group, INS-1 cells treated with high glucose for 48 h significantly activated the NLRP3 and GSDMD expression both at protein and mRNA levels (all *P* < 0.01), whereas EMD treatment (20 *μ*M) significantly downregulated the expression of NLRP3 and GSDMD in both protein and mRNA levels (all *P* < 0.05). Collectively, these results suggested that EMD could downregulate NLRP3 and GSDMD expressions in high-glucose-induced INS-1 cells.

### 3.4. EMD Suppressed the High-Glucose-Induced Pyroptosis via NLRP3/GSDMD Signaling

**Figure 6:**
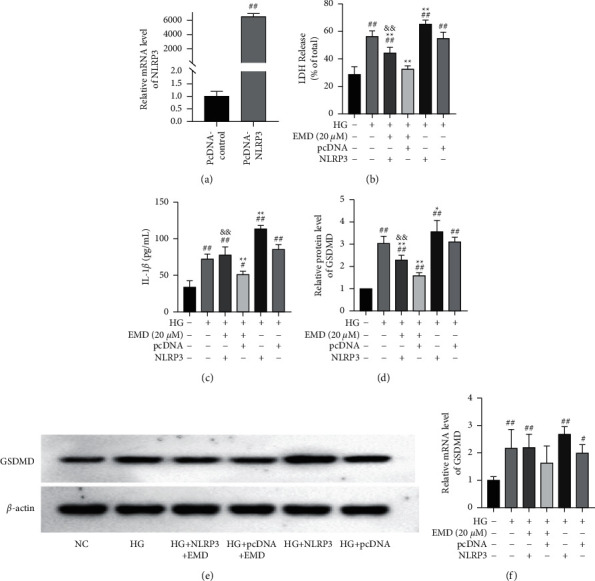
The effects of EMD treatment on the LDH, IL-1*β*, NLRP3, and GSDMD levels induced by high glucose in INS-1 cells after NLRP3 overexpression. (a) NLRP3 mRNA levels are measured by qRT-PCR after cell transfection. (b, c) LDH and IL-1*β* levels are measured by ELISA. (d, e) GSDMD protein levels are measured by western blot. (f) GSDMD mRNA levels are measured by qRT-PCR. Data are expressed as mean ± SD of three independent experiments. ^#^*P* < 0.05 and ^##^*P* < 0.01 vs. the NC group; ^*∗*^*P* < 0.05 and ^*∗∗*^*P* < 0.01 vs. the HG + pcDNA group; ^&^*P* < 0.05 and ^&&^*P* < 0.01 vs. the HG + pcDNA + EMD group.

We further examined the possible molecular mechanism by which EMD suppressed the high-glucose-induced pyroptosis in INS-1 cells. The NLRP3 overexpression plasmid was constructed to treat INS-1 cells. Together with high glucose, the transfection time was set at 24 h before EMD (20 *μ*M) treatment. To determine whether NLRP3 was successfully transfected in INS-1 cells, qRT-PCR was used to measure the mRNA level of NLRP3 (Figure 6(a)). The result showed that compared with the pcDNA-control group, NLRP3 was significantly increased (*P* < 0.01) after transfection in high-glucose-induced INS-1 cells suggesting transfection success.

**Figure 7:**
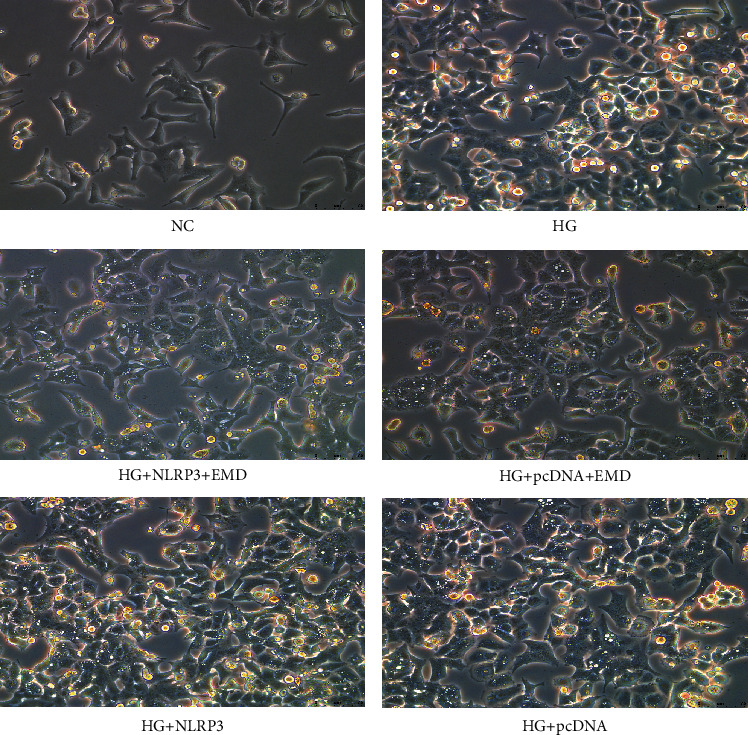
NLRP3 overexpression reversed the effects of EMD treatment on the morphological changes induced by high glucose in INS-1 cells. The morphology was observed under the inverted phase-contrast microscope.

The inverted phase-contrast microscope showed that INS-1 cells' swelling denaturation and small white bubble formation were obvious in the HG group, HG + pcDNA group, and HG + NLRP3 + EMD group, while cell swelling and necrosis were alleviated in the HG + pcDNA + EMD group (Figure 7).

Then, the level of LDH and IL-1*β* was monitored by ELISA (Figures 6(b) and 6(c)), and GSDMD expression was detected by western blot and qRT-PCR in INS-1 cells (Figures 6(d)–6(f)). Combining the former results, high glucose increased the release of LDH, IL-1*β*, and GSDMD protein expression (*P* < 0.01), whereas EMD treatment significantly rescued these changes (all *P* < 0.05). Moreover, overexpression of NLRP3 reversed these effects of EMD treatment on LDH, IL-1*β*, and GSDMD. These results revealed that EMD attenuated high-glucose-induced pyroptosis via the inhibition of NLRP3/GSDMD activation.

Taken together, these results demonstrated that EMD may inhibit NLRP3/GSDMD signaling to prevent the pyroptosis in high-glucose-induced INS-1 cells.

## 4. Discussion

*β*-Cell dysfunction and destruction play a central role in the pathological progress of DM. Despite the current use of oral antidiabetic drugs, insulin therapy, and islet transplant to cure this disease, the outcome faced with inevitable adverse effects was still unsatisfactory. Inflammation and pyroptosis are involved in the pancreatic islet during chronic hyperglycemia, while tailoring innate immunity could prevent DM development [25]. EMD, an anthraquinone compound, exerts potent antidiabetic ability to ameliorate impaired glucose metabolism through the multiple-target mechanism [10]. For *β*-cells, EMD was identified with an indirect regulation by elevation of the glucagon-like peptide-1 level and dipeptidyl peptidase-4 inhibition [12, 13]. The present study demonstrated that EMD could directly attenuate high-glucose-induced *β*-cell inflammation and pyroptosis via inhibiting NLRP3/GSDMD signaling. Thus, we provided a molecular mechanism of EMD treatment in *β*-cell protection and DM prevention.

The production of IL-1*β* was considered as the main cause of early islet destruction during extracellular high glucose. Evidence suggested that the NLRP3 activation could stimulate IL-1*β* maturation and secretion, which regulated the pyroptosis in a wide spectrum of cells [26]. However, whether NLRP3 activation plays a central role in *β*-cell loss remains controversial. Previous studies revealed that deletion or overexpression of NLRP3 had no change in IL-1*β* release and severity of pancreatic injury and could not prevent *β*-cells from pyroptosis during hyperglycemia [27–29]. In contrast, emerging studies reported inhibition of NLRP3 signaling abolished IL-1*β* production and increased *β*-cell vitality [30–32]. A recent study reported that empagliflozin reduced the diabetic pancreatic tissue from hyperglycemia-induced damage of db/db mice via the suppression of the NLRP3/caspase-1/GSDMD pathway [33]. Same results were also achieved in *β*TC-6 cells [33]. Complex mechanism and interaction with its downstream effector and the bias influence of myeloid-derived-IL-1*β* may be the reason why there is a variation in results associated with NLRP3 blockade. In the present study, NLRP3 signaling was found activated by high glucose (50 *μ*M) in vitro, which caused pyroptosis and inflammation in INS-1 cells, whereas EMD treatment protected INS-1 cells against pyroptosis by eliminating the expression of NLRP3, GSDMD cleavage, and IL-1*β* release. Moreover, overexpression of NLRP3 reversed the beneficial effects of EMD. These results fully indicated the molecular mechanism of EMD in *β*-cells via NLRP3/GSDMD signaling.

Pyroptosis is a highly inflammatory form of cell death coupled with DNA fragment, nuclear condensation, cell rupture, water influx, and osmotic lysis, similar to that programmed cell death but not apoptosis [3, 34]. The role of Gasdermin D (GSDMD), an important executor of pyroptosis, is mainly related to the formation of holes in the cell membrane by the N-terminal fragment. [35, 36]. Currently, the main consciousness of GSMD is on the upstream regulators that control GSDMD cleavage. For example, genetic paucity of NLRP3 leads to lower GSDMD cleavage thus inhibiting pyroptosis [37]. Furthermore, different irritants and cell types had different impacts on the cleavage rate of GSDMD [38]. The present study used high glucose to provide a priming and activating signal to activate NLRP3 and observed significant morphological changes under the inverted microscope in INS-1 cells. Correspondingly, the GSDMD expression was increased during hyperglycemia. In addition, EMD treatment was confirmed to rescue normal shape and function of INS-1 cells during hyperglycemia via the inhibition of NLRP3/GSDMD signaling, suggesting EMD could regulate the upstream of GSDMD cleavage and suppress pyroptosis. Interestingly, downstream factors of GSDMD which can enhance the pore formation after GSDMD cleavage also promoted pyroptosis. A current study revealed that the Regulator-Rag complex, a mediator of mTOR activities, was necessary for the GSDMD pore-forming activity and oligomerization in macrophages [35], but whether EMD could suppress the downstream factors of GSDMD in INS-1 cells remains a question for further study. In addition, our in vitro study certainly has some limitations as DM is a complex disease. We will conduct in vivo studies in the future to provide more evidence for the protective effect of EMD through inhibiting pyroptosis in pancreatic islets.

Collectively, this study showed EMD exhibited a potent antipyroptotic effect in INS-1 cells by regulating the upstream of GSDMD.

## 5. Conclusion

In conclusion, our study indicated that EMD had a potent effect against the high-glucose-induced pyroptosis in INS-1 cells by inhibiting NLRP3/GSDMD signaling. Our findings provide a novel mechanism to warrant further investigation for the treatment of EMD as a therapeutic agent against *β*-cell destruction.

## Data Availability

The datasets used and/or analyzed in this study are available from the corresponding author upon reasonable request.
